# Intensive exercise therapy for restenosis after superficial femoral artery stenting: the REASON randomized clinical trial

**DOI:** 10.1007/s00380-022-02060-9

**Published:** 2022-04-09

**Authors:** Tamon Kato, Takashi Miura, Shuhei Yamamoto, Yusuke Miyashita, Naoto Hashizume, Kyoko Shoin, Shinya Sasaki, Yusuke Kanzaki, Hisanori Yui, Shusaku Maruyama, Ayumu Nagae, Takahiro Sakai, Tatsuya Saigusa, Soichiro Ebisawa, Ayako Okada, Hirohiko Motoki, Uichi Ikeda, Koichiro Kuwahara, The REASON Investigators, Tamon Kato, Takashi Miura, Shuhei Yamamoto, Yusuke Miyashita, Naoto Hashizume, Kyoko Shoin, Shinya Sasaki, Yusuke Kanzaki, Uichi Ikeda

**Affiliations:** 1 Department of Cardiovascular Medicine, Shinshu University Hospital, Shinshu University School of Medicine, 3-1-1 Ashahi, Matsumoto, 390-8621 Japan; 2 Department of Cardiology, Nagano Municipal Hospital, Nagano, Japan; 3 Department of Rehabilitation, Shinshu University Hospital, Matsumoto, Japan; 4 Department of Cardiology, Nagano Red Cross Hospital, Nagano, Japan; 5 Department of Cardiology, Shinonoi General Hospital, Nagano, Japan; 6 Department of Cardiology, Aizawa Hospital, Matsumoto, Japan; 7 Department of Cardiology, Saka General Hospital, Tagajyo, Japan

**Keywords:** Exercise, In-stent restenosis, Endovascular therapy, Peripheral artery disease, Femoropopliteal lesions

## Abstract

Endovascular treatment (EVT) is the main treatment for peripheral artery disease (PAD). Despite advances in device development, the restenosis rate remains high in patients with femoropopliteal lesions (FP). This study aimed to evaluate the effectiveness of exercise training in reducing the 1-year in-stent restenosis rate of bare metal nitinol stents for FPs. This prospective, randomized, open-label, multicenter study was conducted from January 2017 to March 2019. We randomized 44 patients who had claudication with de novo stenosis or occlusion of the FP into an intensive exercise group (*n* = 22) and non-intensive exercise group (*n* = 22). Non-intensive exercise was defined as walking for less than 30 min per session, fewer than three times a week. We assessed exercise tolerance using an activity meter at 1, 3, 6, and 12 months, and physiotherapists ensured maintenance of exercise quality every month. The primary endpoint was instant restenosis defined as a peak systolic velocity ratio > 2.5 on duplex ultrasound imaging. Kaplan–Meier analysis was used to evaluate the data. There were no significant differences in background characteristics between the groups. Six patients dropped out of the study within 1 year. In terms of the primary endpoint, intensive exercise significantly improved the patency rate of bare nitinol stents at 12 months. The 1-year freedom from in-stent restenosis rates were 81.3% in the intensive exercise group and 47.6% in the non-intensive exercise group (*p* = 0.043). No cases of stent fracture were observed in the intensive exercise group. Intensive exercise is safe and reduces in-stent restenosis in FP lesions after endovascular therapy for PAD. Clinical trial registration: University Hospital Medical Information Network Clinical Trials Registry (No. UMIN 000025259).

## Introduction

The prevalence of peripheral artery disease (PAD) is approximately 5% in the general population aged > 40 years and increases to approximately 15% among adults aged > 70 years [1]. Femoropopliteal (FP) lesions are present in 60–70% of patients with symptomatic PAD [2–4]. Although endovascular therapy (EVT) is the first-line treatment for FP lesions, the yearly incidence of restenosis for bare metal nitinol stents (BNS) is 20–50% [5–8]. Cilostazol reduces the in-stent restenosis (ISR) rate of BNS by almost half by increasing blood flow in the limbs [9, 10]. Supervised exercise training (SET) is strongly recommended as the initial treatment modality for patients with intermittent claudication, as it increases blood flow in the limbs, similar to cilostazol [11]. However, supervised exercise is limiting for patients with PAD because it requires regular transportation to the exercise center.

A home-based walking exercise intervention is sometimes reported as a promising alternative to SET [12]. We hypothesized that such an intervention would reduce the ISR rate of BNS for FP lesions. Thus, we designed the Intensive Exercise Therapy for Restenosis after Superficial Femoral Artery Stenting (REASON) randomized clinical trial to evaluate the effectiveness of exercise training in reducing the 1-year ISR rate of BNS for FP lesions.

## Materials and methods

### Ethics statements

Written informed consent was obtained from all patients. The present study was approved by the ethics committees of each hospital and was performed in accordance with the Declaration of Helsinki. The study was registered with the University Hospital Medical Information Network Clinical Trials Registry, as accepted by the International Committee of Medical Journal Editors.

### Study design and population

This was a prospective, randomized, open-label, multicenter study. From January 2017 to March 2019, 44 patients with symptomatic PAD due to de novo FP lesions from six cardiovascular centers were enrolled and randomized.

All patients with PAD and symptomatic claudication or resting pain (Rutherford classes 2–4) who had de novo FP lesions and were referred to the outpatient clinic at participating hospitals were eligible. Patients who were unable to walk, those with FP lesions involving inflow aortoiliac lesions, those with poor below-the-knee runoff (defined as < 1 below-the-knee runoff), and those with acute or sub-acute limb ischemia were excluded.

### Randomization

Eligible patients were assigned in a 1:1 ratio to either a combination of supervised and home-based exercise or an attention control condition. Randomization was performed centrally using a computerized algorithm.

### Exercise program

The participants underwent supervised exercise by trained physiotherapists in their own hospital once a month. The exercise program consisted primarily of treadmill walking to near-maximum claudication pain. Participants were advised to walk to until they experienced severe discomfort and to resume walking once it had subsided sufficiently. At that time, they agreed to stop smoking and to continue their diet and home-based exercise. For the home-based exercise regimen, participants were advised to start with a frequency of at least three sessions every week and approximately 30–45 min per session. Participants without leg symptoms were asked to walk to an intensity of 12–14 on the Borg Rating of Perceived Exertion scale.

### Endovascular procedure and lesion measurement

All patients were pretreated with clopidogrel (75 mg daily), in addition to aspirin (100 mg daily). Anti-platelet therapy was started at least 2 days before the procedure and continued as dual antiplatelet therapy for at least 1 month. The approach site was determined at the discretion of the physicians. In almost all cases, EVT was performed using a 6-French sheath via the contralateral or ipsilateral femoral artery. After infusing 3000–5000 U of heparin, the lesion was crossed with a 0.014- or 0.035-inch guidewire. The lesion was dilated with a semi-compliant, non-compliant, or scoring balloon, which was 1 mm smaller than the diameter of the distal vessel before stent implantation. The patients received a self-expandable nitinol stent. After implanting the stent, which had a diameter 1 mm larger than the reference vessel, post-dilatation was performed with a non-compliant balloon whose size was equivalent to the diameter of the distal vessel. The decision to use intravascular ultrasonography was performed at the discretion of the physicians. The proximal and distal reference vessels and lesion length were measured from angiographic data.

### Outcome assessment and data collection

Each patient was assessed for symptoms, the ankle–brachial index (ABI), and presence of restenosis using duplex ultrasonography at the 1-, 3-, 6-, and 12-month follow-up visits. At 12 months after EVT, patients who were alive without any reintervention and restenosis were regularly scheduled to walk for 6 min. Baseline medical history and demographic data, including age and sex, were obtained from the patients’ reports to allow external generalizability of the study results. Blood examination data, weight, and height were measured during the study visits. The primary outcome measure was 1-year restenosis based on duplex ultrasonography findings (peak systolic velocity ratio > 2.5). All stent lengths were measured to determine whether one or multiple stents were placed. The secondary outcome measure was the occurrence of major adverse limb events (MALEs), defined as a composite of limb-related death, target lesion revascularization (TLR), major amputation, major bleeding, and definite or probable stent thrombosis.

### Statistical analysis

The sample size was estimated based on the binary restenosis rates from previous trials with cilostazol, based on the assumption that exercise exhibits a similar ability to reduce ISR when compared with cilostazol. An overall sample size of 180 patients would be required to detect a difference in 1-year ISR after EVT with intensive exercise when compared with non-intensive exercise at a power of 80% and 2-sided α of 0.05, assuming binary restenosis rates of 20% in the EVT with intensive exercise and cilostazol group and 50% in the EVT without intensive exercise group. We evaluated the primary and secondary endpoints at one year using the Kaplan–Meier method.

Data are shown as the mean ± standard deviation for continuous variables or as percentages for dichotomous variables, unless specified otherwise. Baseline medical history and demographic data for the two groups were compared while correcting for multiple comparisons. All results were based on intention-to-treat analysis. For the primary outcome measure, life-table analysis using fixed time points of observation was performed to assess the differences in primary patency between the two groups. Statistical analyses were performed using SPSS version 24 (IBM Corp., Armonk, NY, USA). Statistical significance was set at *p* < 0.05.

## Results

**Fig. 1:**
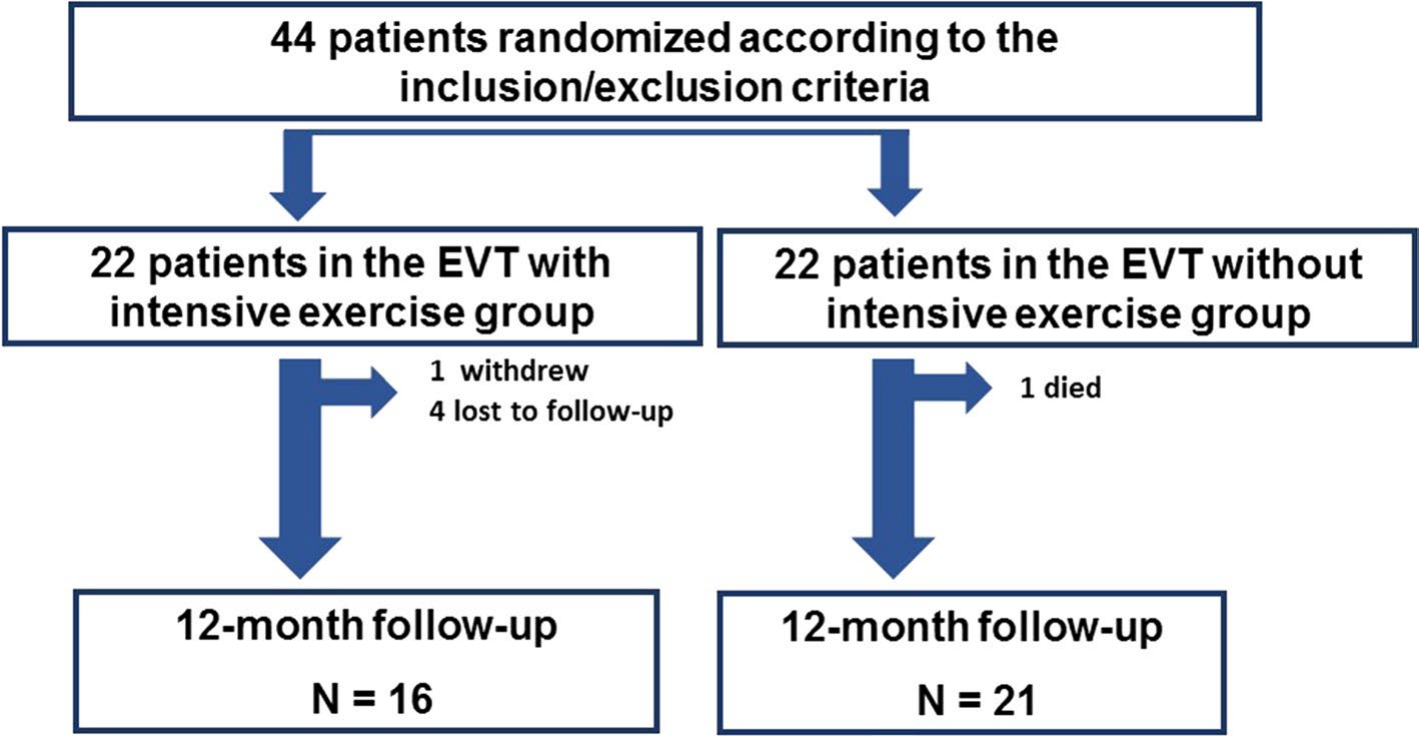
Patient flow chart. Eligible patients were assigned in a 1:1 ratio to either a combination of supervised and home-based exercise or an attention control condition. In the EVT with intensive exercise group, one patient withdrew from the study, and four patients were lost before follow-up. One patient in the EVT with no exercise group died. Finally, we analyzed data for 16 patients in the intensive exercise group and 21 patients in the non-intensive exercise group. *EVT* endovascular therapy

Forty-four patients were randomly assigned to either EVT with intensive exercise (intensive exercise group, *n* = 22) or EVT without intensive exercise (non-intensive exercise group, *n* = 22). Five patients in the intensive exercise group were lost to follow-up (four lacking clinical data and one who had withdrawn from the study), and one patient in the non-intensive exercise group died. The causes of data loss were patient-related technical concerns, such as patients forgetting to wear the meter or turn the device on. Finally, we evaluated data for 16 patients in the intensive exercise group and 21 patients in the non-intensive exercise group (Fig. 1).

**Fig. 2:**
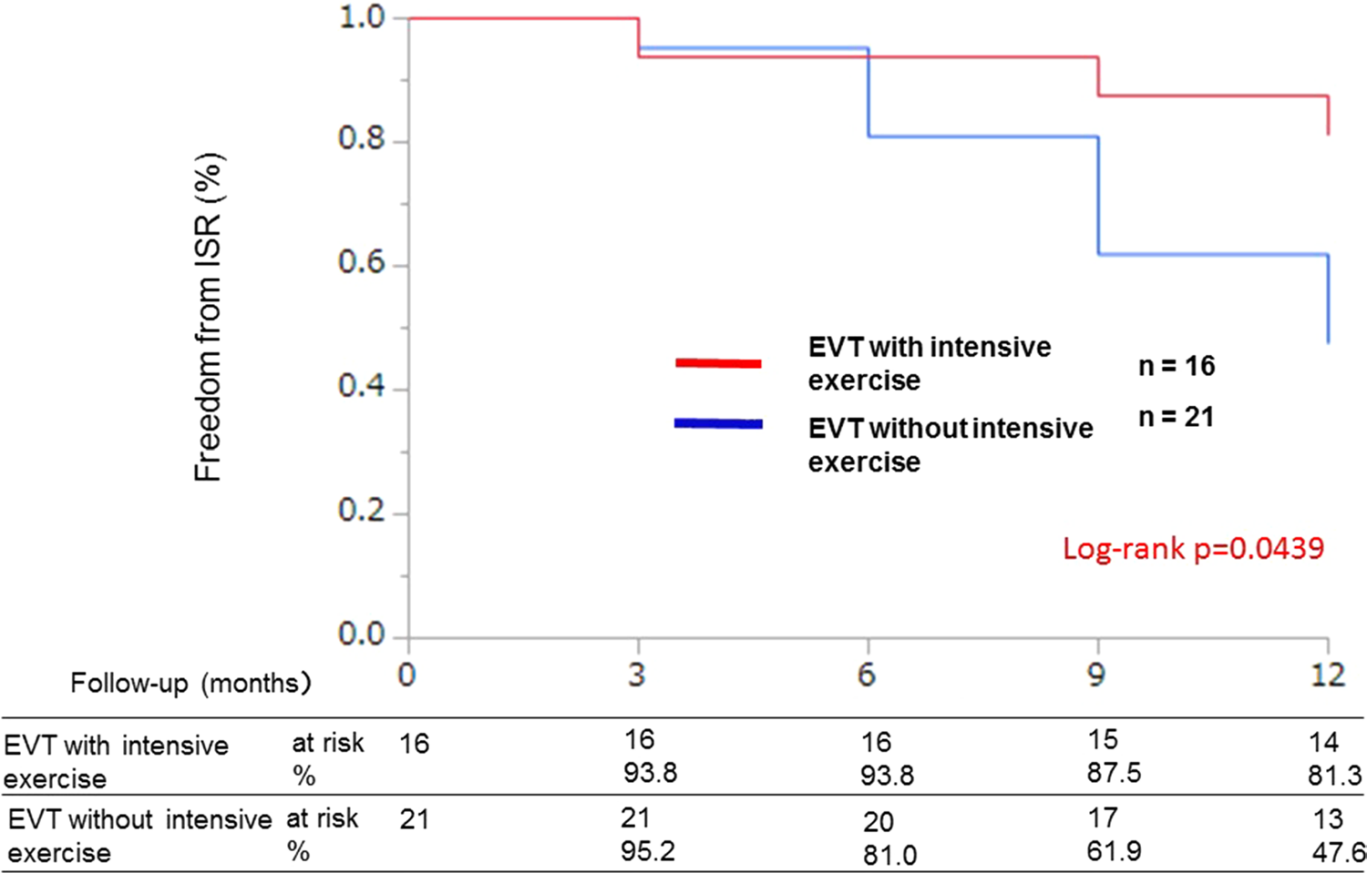
Kaplan–Meier graph of freedom from TLR at 1 year. Kaplan–Meier analysis indicated that the rate of freedom from ISR in the EVT with intensive exercise group was higher than that in the EVT without intensive exercise group. *EVT* endovascular therapy, *TLR* target lesion revascularization, *ISR* in-stent restenosis

**Fig. 3:**
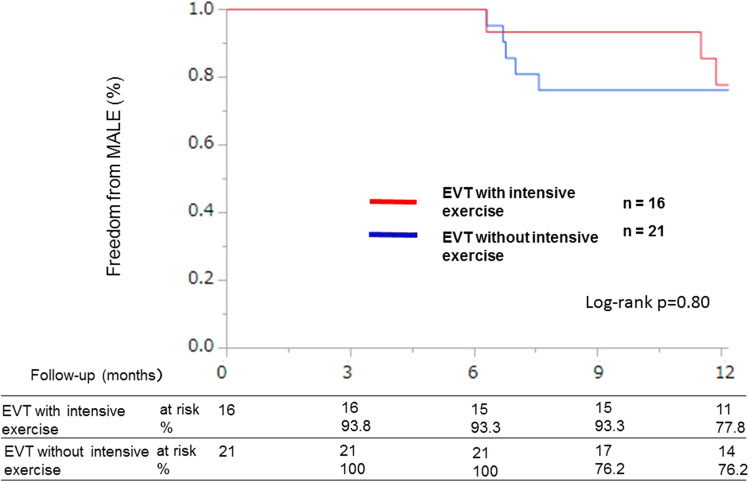
Kaplan–Meier graph of freedom from MALEs at 1 year. There was no significant difference in MALEs between the intensive exercise and non-intensive exercise groups. *MALE* major adverse limb event (limb-related death, target lesion revascularization, major amputation, major bleeding, and definite or probable stent thrombosis), *EVT* endovascular therapy, *ISR* in-stent restenosis

The two groups were well matched at baseline in terms of demographic data such as age (intensive exercise group: 72.6 ± 6.8 vs. non-intensive exercise group: 74.5 ± 8.5 *p* = 0.471), diabetes mellitus (56.3% vs. 52.4% *p* = 0.890), estimated glomerular filtration rate (eGFR) (66.26 [48.1, 81.8] vs. 68.6 [53.2, 72.7] *p* = 0.859), C-reactive protein level (0.06 [0.04, 0.20] vs. 0.11 [0.06, 0.21] *p* = 0.44), 6-min walking distance results (367 [321, 416] vs. 360 [274, 405] *p* = 0.58) (Table 1), and lesion characteristics (Table 2). The median length of the treated segment was 80 mm (interquartile range, 27.5, 205.0 mm), and 47.4% were chronic total occlusion lesions. Atlantic Inter-Society Consensus (TASC) II class C/D lesions were present in 38.9% of patients in the intensive exercise group and 26.1% of patients in the non-intensive exercise group. The rates of freedom from ISR at 1 year were 81.3% in the intensive exercise group and 47.6% in the non-intensive exercise group (log-rank *p* = 0.043, Fig. 2). There was no significant difference in the freedom from MALEs between the intensive and non-intensive exercise groups (77.8% and 76.2%, respectively; log-rank *p* = 0.80) (Fig. 3). There were no cases of major amputation, onset of critical limb ischemia, or accidents during exercise. There were only two stent fractures in the non-supervised exercise treatment group.

**Table 1:** Baseline characteristics

Variables	EVT with intensive exercise (*n* = 16)	EVT without intensive exercise (*n* = 21)	*p* value
Age (years)	72.6 ± 6.8	74.5 ± 8.5	0.471
Male	11 (68.8)	16 (76.2)	0.790
Body mass index (kg/m^2^)	23.2 [21.5, 25.3]	22.2 [19.4, 26.1]	0.329
Hypertension	15 (93.8)	19 (90.5)	0.951
Dyslipidemia	9 (56.3)	13 (61.9)	0.678
Diabetes mellitus	9 (56.3)	11 (52.4)	0.890
Insulin use	2 (12.5)	4 (19.0)	0.572
Current smoker	4 (25.0)	6 (28.6)	0.775
Previous smoker	11 (68.8)	16 (76.2)	0.571
Hemodialysis	2 (12.5)	2 (9.5)	0.796
Previous stroke	2 (12.5)	4 (19.0)	0.572
CAD	9 (56.3)	15 (71.4)	0.326
Previous heart failure	1 (6.3)	1 (4.8)	0.859
Rutherford class II	2 (12.5)	4 (19.0%)	0.34
III	13 (81.3)	16 (76.2)	0.711
IV	1 (6.3)	1 (4.8)	0.84
LV dysfunction	0 (0.0)	1 (4.8)	0.370
LVEF	66 [53.5,74.5]	63.5 [56.7, 70.2]	0.86
ABI	0.72 [0.56, .88]	0.65 [0.56, .82]	0.85
6-min walking distance (m)	367 [321, 416]	360 [274, 405]	0.58
HbA1c	6.1 [5.6, 7.4]	6.4 [6.2, 7.5]	0.12
eGFR (mL/min/1.73 m^2^)	66.26 [48.1, 81.8]	68.6 [53.2, 72.7]	0.859
LDL-C	107 [69, 118.5]	80 [70, 101]	0.23
HDL-C	50 [38, 64]	47 [45.8, 58]	0.54
TG	112 [93, 200]	120 [82.3, 155.3]	0.52
CRP	0.06 [0.04, 0.20]	0.11 [0.06, 0.21]	0.44
Cilostazol	5 (31.3)	8 (38.1)	0.666
Statins	10 (62.5)	16 (76.2)	0.503
ACE I/ARBs	9 (56.3)	9 (42.9)	0.350
β-Blockers	5 (31.3)	9 (42.9)	0.542
Ca-antagonists	10 (62.5)	14 (66.7)	0.943

Data are shown as the mean ± SD, median [interquartile range], or *n* (percentage)

*EVT* endovascular therapy, *CAD* coronary artery disease, *LVEF* left ventricular ejection fraction, *ABI* ankle–brachial index, *eGFR* estimated glomerular filtration rate, *LDL-C* low-density lipoprotein cholesterol, *HDL-C* high-density lipoprotein cholesterol, *TG* triglyceride, *ACE-I* angiotensin-converting-enzyme inhibitor, *ARB* angiotensin II receptor blocker

**Table 2:** Lesion characteristics

Variables	EVT with intensive exercise (*n* = 16)	EVT without intensive exercise (*n* = 21)	*p* value
TASC IIC/D	7 (43.8)	6 (28.6)	0.290
CTO	8 (50.0)	9 (42.9)	0.578
Calcification	2 (12.5)	13 (61.9)	0.004
Lesion length (mm)	100.0 [35.0, 212.5]	65.0 [28.8, 277.0]	0.625
Distal diameter of reference vessel (mm)	6.0 [6.0, 6.0]	5.8 [5.0, 6.0]	0.748
Number of below-the-knee runoffs, 1/2/3	2/11/3	8/6/7	0.024
Number of stents			
1	10 (62.5)	16 (76.2)	0.48
2	6 (37.5)	4 (19.0)	0.75
3	0 (0.0)	1 (4.8)	0.76

Data are shown as the mean ± SD, median [interquartile range], *n* (percentage), or number per group (*n*/*n*/*n*)

*EVT* endovascular therapy, *TASC* Trans-Atlantic Inter-Society Consensus, *CTO* chronic total occlusion

In terms of secondary endpoints, the rates of TLR (85.6% and 75.0%, respectively; *p* = 0.87), major amputation (0% and 0%, respectively), major bleeding (7.7% and 0%, respectively, *p* = 0.12), and stent thrombosis (0% and 0%, respectively) were similar between the intensive and non-intensive exercise groups. In the intensive exercise group, there were two cases of Tosaka class III restenosis (12.5%) and one case of Tosaka class I restenosis (6.25%). In the non-intensive exercise group, there were four cases of Tosaka class I restenosis (19.5%), five cases of Tosaka class II restenosis (23.8%), and two cases of Tosaka class III restenosis (9.5%). There was no significant difference in the distribution of ISR type between the groups (*p* = 0.08).

Median step and caloric data from the activity tracker in the intensive exercise group were as follows: steps, 2868 [1640–4917]; total calories, 1899 [1642–2111]; and exercise calories, 394 [307–576]/month. Both groups exhibited an improvement in Rutherford classification, but no significant difference was observed between the two groups. There was no significant difference in 6-min walking distance at baseline or 1 year later between the intensive and non-intensive exercise groups (baseline: 367 [279–405] and 363 [318–419], *p* = 0.77; 1 year: 377 [343–417] and 350 [307–481], *p* = 0.80, respectively). Furthermore, there was no significant difference in ABI at baseline or 1 year later between the intensive and non-intensive exercise groups (baseline: 0.95 [0.9–1.08] and 1.0 [0.96–1.1], *p* = 0.3; 1 year: 0.93 [0.78–1.0] and 0.96 [0.66–1.06], *p* = 0.82, respectively) (Table 3). However, there was a correlation between calorie intake and 6-min walking distance (*r* = 0.58, *p* = 0.047), and between the number of steps and ABI at 1 year (*r* = 0.61, *p* = 0.027).

**Table 3:** Characteristics of the two groups at the 1-year follow-up

Variables	EVT with intensive exercise (*n* = 16)	EVT without intensive exercise (*n* = 21)	*p* value
Body mass index (kg/m^2^)	23.9 [21.6, 25.8]	23.5 [19.9, 25.6]	0.56
LVEF	64.0 [51.0–68.5]	64.7 [46.9–68.3]	0.72
Rutherford class I	2 (12.5)	1(4.8)	0.28
II	6 (37.5)	8 (38.1)	0.92
III	8 (50.0)	12 (57.1)	0.78
IV	0	0	
ABI	0.95 [0.70, 1.05]	0.92 [0.78,1.0]	0.90
6-min walking distance	380 [310, 450]	374 [339, 397]	0.88
Difference in 6-min walking distance	17 [− 40, 45]	− 11 [− 40.5, 36.8]	0.56
HbA1c	6.4 [5.8–7]	6.7 [6.2–6.8]	0.39
eGFR	63 [51, 69]	66 [54.5, 74]	0.65
TC	168.0 [144.5, 220.5]	151.5 [129.3, 184.5]	0.37
LDL-C	100 [81.5–134.5]	76.5 [59.0, 96.3]	0.59
HDL-C	48.0 [44.0–57.0]	49.0 [40.5–57.3]	0.86
TG	110.5 [71.3, 161.8]	107.0 [93.5, 146.5]	0.98
CRP	0.05 [0.03–0.2]	0.07 [0.04–0.2]	0.30

*EVT* endovascular therapy, *LVEF* left ventricular ejection fraction, *ABI* ankle–brachial index, *eGFR* estimated glomerular filtration rate, *TC* total cholesterol, *LDL-C* low-density lipoprotein cholesterol, *HDL-C* high-density lipoprotein cholesterol, *TG* triglyceride, *ACE-I* angiotensin-converting-enzyme inhibitor, *ARB* angiotensin II receptor blocker

## Discussion

### Main findings

To the best of our knowledge, the REASON study is the first to reveal that intensive exercise significantly improves the patency rate of BNS at 12 months, thus confirming our hypothesis. Furthermore, there were no cases of stent fracture at the 12-month follow-up in patients who performed intensive exercise, and our findings indicated a relationship between exercise strength and 6-min walk distance at 1 year in the intensive exercise group. We also observed no significant differences in the 6-min walking distance or ABI at 1 year between the two groups.

In patients with PAD exhibiting claudication, SET is recommended to improve functional status and quality of life (QoL) and reduce leg symptoms [13, 14]. In a meta-analysis of 15 randomized controlled trials including 1257 patients treated with SET or optimal medical therapy, SET was found to significantly improve health-related QoL [15]. The Claudication: Exercise Versus Endoluminal Revascularization study randomized patients with symptomatic aortoiliac PAD and reported comparable benefits for SET and stent revascularization at 6–18 months [16, 17].

EVT has been established in recent years as the first choice to treat occlusive disease in the FP arteries. It is less invasive than surgical approaches, and the evolution of endovascular techniques and devices has made it more efficient. According to TASC II, the outcome of EVT depends on clinical and anatomical factors including diabetes mellitus, renal failure (hemodialysis), critical limb ischemia, total lesion length, and severity of the lesion in the outflow arteries (poor runoff) [18]. Cilostazol reduces ISR after FP stenting, and its intake is recommended according to the guidelines [9, 10]. Since the clinical outcome of FP stenting is not satisfactory, drug-eluting technologies have been developed to improve the patency rate by inhibiting neointimal hyperplasia and smooth muscle cell proliferation. We believe that the effect of exercise on clinical outcomes was not influenced by the revascularization strategy and device, but the effect of exercise when combined with drug technology should be considered. In the present study, intensive exercise was supervised monthly using an activity tracker. Supervised exercise was effective, but it was very difficult for patients to perform the exercise strictly. Nonetheless, even non-strict adherence to the regimen exerted some effect on improving clinical outcomes.

### Safety endpoint

In this study, there were no cases of major amputation or onset of critical limb ischemia. There were also no accidents associated with exercise. Usually, there is a considerable risk of stent fracture after femoral artery lesions, which is associated with higher ISR and re-occlusion rates [19]. However, in our study, there were only two stent fractures in the non-intensive exercise group. There were three cases of stenting in the P1 segment, and there were no stent fractures in the intensive exercise group, suggesting that our intensive exercise regimen was safe and did not cause adverse events or accidents during the study period.

### Type of restenosis

Intensive exercise did not influence the type of restenosis in this study. ISR or re-occlusion is a very complex phenomenon that consists of increased neointima and thrombi.

### Exercise strength

Calorie intake was correlated with 6-min walking distance at 1 year, while step counts were correlated with the ABI at 1 year. This result suggests that exercise strength may affect 1-year clinical outcomes in patients with intermittent claudication. In this study, calorie intake exhibited a stronger correlation with 6-min walking distance than step count, suggesting that the intensity of exercise is more important than the amount for improving 6-min walking distance.

### Mechanism underlying the suppression of in-stent restenosis and re-occlusion

In this study, we did not collect data related to the mechanism of stent restenosis. However, the clinical effects of exercise are well established. Pre-clinical and multiple human investigations have highlighted several mechanisms for such clinical benefits, including improvements in endothelial function [20] and the inflammatory response. During exercise training, increased blood flow and shear stress improve vascular hemostasis by reducing the production of reactive oxygen species and increasing the availability of nitric oxide in the endothelium [20]. The reduction in inflammatory/hemostatic markers associated with higher levels of physical activity accounts for a major portion of the reduced risk of cardiovascular disease in individuals [21].

Given the current lack of evidence supporting exercise therapy for the population with PAD, including those with intermittent claudication as well as those with chronic limb-threatening ischemia, further evidence is required before reimbursement policies for supervised exercise can be reconsidered.

### Limitations

There are several limitations to the current study. First, although a randomized control design was used, our sample size was small. Although the sample size was initially set to 240 cases, fewer patients could be registered due to the strict nature of the intensive exercise protocol. This small sample size also prevented us from comparing predictors between the present and previous studies. Second, there may have been selection bias during patient enrollment, which was performed by the attending physician. While we attempted to register all cases, those registered were not consecutive cases. Third, we did not provide activity monitors to patients in the non-intensive exercise group given the difference in the amount of home-based exercise between the two study groups. Thus, we were unable to compare the amount of activity between the intensive group and the non-intensive group, resulting in a large limitation. Fourth, we only analyzed data for patients with BNS. Further studies of new devices, such as drug-eluting stents and drug-coated balloons, are required. Fourth, we could not evaluate the quality or amount of exercise in patients who did not undergo SET. Finally, the patients in this study were Japanese, and their physical constitution and muscle strength may differ from those of Western patients.

In conclusion, our findings indicated that intensive exercise therapy did not increase rates of stent fracture and reduced the 1-year ISR in FP lesions after EVT for PAD. To the best of our knowledge, the REASON study is the first to reveal that intensive exercise can safely and effectively improve the patency rate of BNS at 12 months in this population.
